# Differences in Time to Clinical Response to Tezepelumab between Th2-high and Th2-low Phenotypes in Severe Asthma: A Real-world Analysis

**DOI:** 10.31662/jmaj.2026-0164

**Published:** 2026-08-21

**Authors:** Hideyasu Yamada, Taisuke Nakaizumi

**Affiliations:** 1 Kagurazaka Allergy and Snoring Clinic, Tokyo, Japan; 2 Hitachinaka General Hospital, Ibaraki, Japan

**Keywords:** tezepelumab, severe asthma, phenotype, time to response, thymic stromal lymphopoietin (TSLP)

Severe asthma is a highly heterogeneous disease driven by complex, overlapping inflammatory pathways. Recently, the pathophysiological paradigm has shifted from downstream effector cells to upstream airway epithelium-derived alarmins, particularly thymic stromal lymphopoietin (TSLP) ^(1)^. Tezepelumab, a first-in-class human monoclonal antibody targeting TSLP, has indicated broad and potent efficacy in reducing asthma exacerbations and improving lung function, irrespective of baseline Th2 inflammatory biomarker levels ^(2)^.

In real-world clinical practice, the timely evaluation of a biologic’s effectiveness is critical for optimizing both patient outcomes and health care resources ^(3)^. Current clinical guidelines generally recommend assessing the initial response to biological therapies 12-16 weeks (3-4 months) after initiation ^(4)^. However, because tezepelumab acts at the apex of the inflammatory cascade, the mechanisms and time required to achieve clinically meaningful improvements in symptom control may differ fundamentally between patients with strong baseline type 2 inflammation (Th2-high) and those without such predominant pathways (Th2-low) ^(5)^. We hypothesized that although tezepelumab is ultimately effective for both phenotypes, the temporal trajectory of the clinical response might diverge.

Therefore, this retrospective multicenter observational study aimed to compare the time required to achieve a minimal clinically important difference (MCID) in the Asthma Control Test (ACT) score between Th2-high and Th2-low phenotypes in patients with severe asthma treated with tezepelumab.

This retrospective multicenter observational study included 45 consecutive patients with severe, uncontrolled asthma (Global Initiative for Asthma Step 5) in whom tezepelumab therapy (210 mg administered subcutaneously every 4 weeks) was initiated between April 2023 and March 2026 at two participating institutions (Kagurazaka Allergy and Snoring Clinic and Hitachinaka General Hospital).

To comprehensively evaluate the treatment dynamics and eliminate selection bias, the analysis included all patients, and those who discontinued therapy within 12 months were treated as censored cases. Furthermore, to eliminate the potential confounding effect of prior biomarker suppression, the cohort exclusively comprised patients who were biologic-naïve receiving tezepelumab as their first biologic therapy.

Before tezepelumab initiation, patients were stratified into two groups on the basis of baseline biomarkers: the Th2-high group (n = 24), which met at least one of the criteria (blood eosinophil count ≥300/μL, fractional exhaled nitric oxide [FeNO] ≥25 ppb, or total IgE ≥173 IU/mL [the upper limit of the normal reference range established by the central clinical laboratory, SRL, Inc., Tokyo, Japan]); and the Th2-low group (n = 21), which met none of the previously mentioned criteria.

The primary outcome was the time (measured in months) from the initiation of tezepelumab to the achievement of a clinical response, defined as an improvement in the ACT score of ≥ 3 points from baseline ^(6)^. Secondary outcomes included the evaluation of sustained asthma control based on the maintenance of ACT scores (≥20 points) and the avoidance of maintenance oral corticosteroids (mOCS) bursts at the 12-month follow-up. Importantly, patients who discontinued tezepelumab or switched to another biologic owing to insufficient efficacy before 12 months were classified as having treatment failure for this secondary outcome.

Continuous variables were compared using the Mann-Whitney U test or Welch’s t-test, and categorical variables were analyzed using Fisher’s exact test. The time to clinical response was analyzed using the Kaplan-Meier method, and differences between the Th2 phenotypes were compared using the Log-rank test. Moreover, to rigorously account for the competing risk of treatment discontinuation or switching to another biologic owing to insufficient efficacy, a Fine-Gray subdistribution hazard model was applied for the evaluation of effectiveness, and cumulative incidence curves were estimated using the Aalen-Johansen estimator. A p-value of <0.05 was considered statistically significant. This study was conducted in accordance with the Declaration of Helsinki. All participants provided written informed consent, and the local institutional review board approved the study protocols (institutional review board number: R01-350).

Regarding baseline characteristics (Table 1), there were no significant differences between the Th2-high and Th2-low groups in age (p = 0.50), sex (p = 0.75), and exacerbation history in the previous year (p = 0.88). Crucially, the baseline ACT scores were comparable in the two groups (mean 17.3 in Th2-high vs. 18.8 in Th2-low; p = non-significant), indicating a similar degree of baseline symptom impairment. Regarding mOCS, the proportion of patients requiring mOCS was higher in the Th2-high group than in the Th2-low group.

**Table 1.** Baseline demographic and clinical characteristics of the study patients.

Characteristic	All Patients (n=36)	Th2-High (n=20)	Th2-Low (n=16)	P-value
Age, years (mean)	55.8	57.7	53.4	ns
Sex (Female), n (%)	16 (44.4%)	9 (45.0%)	7 (43.8%)	ns
Exacerbations in past year (mean)	1.6	1.6	1.6	ns
Baseline ACT score (mean)	18.0	17.3	18.8	ns
Blood Eosinophils, /μL (median)	200	324	170	< 0.001
Total lgE, IU/mL (median)	175	300	122	< 0.001
FeNO (median)*	22	35	16.5	< 0.001
mOCS use, n (%)	7 (19.4%)	6 (30.0%)	1 (6.3%)	0.104
mOCS dose, mg/day (median)	0	0	0	-

Values are presented as mean, median, or n (%) as indicated. *Data for FeNO are based on cases where measurement was performed (n = 34; 2 missing in the Th2-high group). ACT: Asthma Control Test; FeNO: fractional exhaled nitric oxide; mOCS: maintenance oral corticosteroids; ns: not significant.

During the 12-month observation period, nine patients (4 in the Th2-high group and five in the Th2-low group) discontinued therapy and were treated as censored cases (6 patients switched to another biologic owing to insufficient efficacy, and three patients terminated treatment).

Kaplan-Meier analysis revealed that the probability of achieving an early clinical response was significantly lower in the Th2-low group than in the Th2-high group (log-rank test, p < 0.01) (Figure 1). For those in whom the clinical response was achieved, the median time to achieve an ACT improvement of ≥3 points was 2.0 months in the Th2-high group. In stark contrast, the median time to response in the Th2-low group was 4.5 months. Notably, some patients with Th2-low required up to 6 months for the MCID criteria to be achieved.

**Figure 1.**
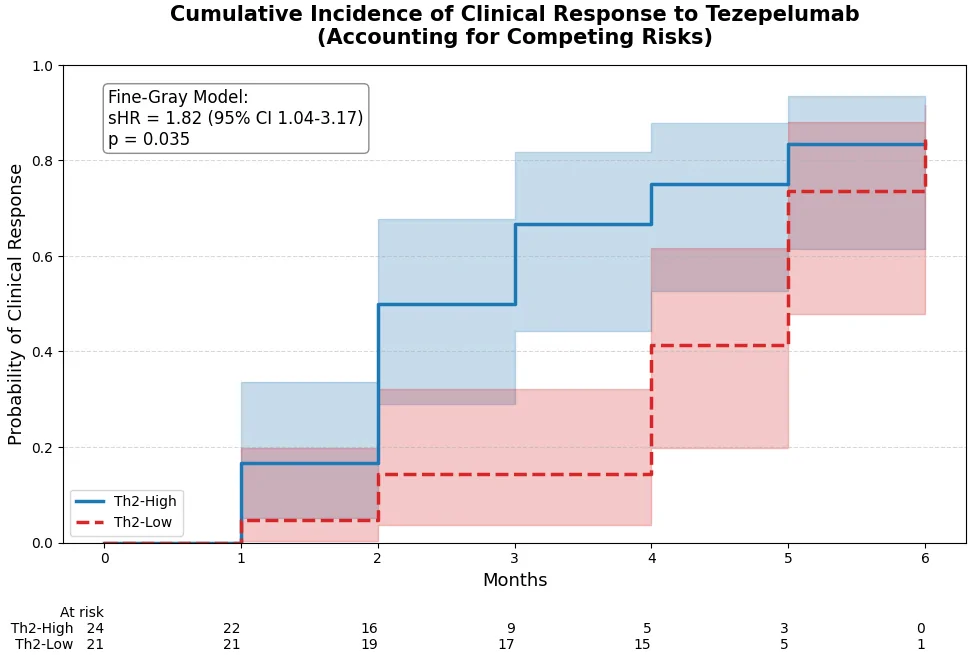
Cumulative incidence of clinical response to tezepelumab by Th2 phenotype. The cumulative incidence curves illustrate the probability of achieving a clinical response over time (months) for the Th2-high (blue solid line) and Th2-low (red dashed line) phenotypes. Discontinuation or switching to another biologic owing to insufficient efficacy was rigorously treated as a competing risk using the Aalen-Johansen estimator. The Fine-Gray subdistribution hazard model revealed that a clinical response was achieved significantly faster in the Th2-high group than in the Th2-low group (sHR, 1.82; 95% CI, 1.04-3.17; p = 0.035). ACT: Asthma Control Test; CI: confidence interval; sHR: subdistribution hazard ratio.

Despite this significant difference in response speed, both groups indicated sufficient and comparable clinical efficacy at the 12-month follow-up, maintaining excellent asthma control without the need for additional mOCS bursts, after appropriately categorizing the six patients who switched to another biologic as having treatment failure.

Although a conventional Kaplan-Meier analysis indicated a stronger statistical significance for the time to clinical response (p < 0.01), we also applied the more conservative Fine-Gray subdistribution hazard model to rigorously account for the competing risk of treatment discontinuation or switching to another biologic owing to insufficient efficacy. Even under this stringent model, the analysis confirmed that the Th2-high phenotype remained significantly associated with an accelerated time to clinical response compared with the Th2-low phenotype. Specifically, the rate of achieving a clinical response was 1.82 times faster in the Th2-high group (subdistribution hazard ratio [sHR], 1.82; 95% confidence interval, 1.04-3.17; p = 0.035).

No severe adverse events were observed throughout the 12-month period.

To our knowledge, this is the first real-world study showing that although tezepelumab is highly effective for both phenotypes, the time to clinical response differs significantly on the basis of the baseline Th2 profile. Because the baseline ACT scores and exacerbation histories were comparable in the groups, the delayed response observed in the Th2-low group cannot be attributed to a milder baseline impairment. Rather, this distinct temporal divergence is likely explained by the mechanistic differences in the underlying pathophysiology ^(7)^.

In Th2-high asthma, the inhibition of TSLP rapidly downregulates downstream type 2 cytokines (interleukin [IL]-5, IL-4/-13), leading to a swift depletion of eosinophilic inflammation and a correspondingly rapid relief of symptoms (median 2.0 months) ^(8)^. Conversely, Th2-low asthma is often driven by non-type 2 mechanisms, including neutrophilic inflammation, mast cell activation, and structural changes such as fixed airway hyperresponsiveness and airway remodeling ^(9)^. Although tezepelumab effectively modulates these non-type 2 pathways, the reversal of tissue-level remodeling inherently entails a longer biological time lag before translating into subjective symptom improvement ^(10)^.

We acknowledge a limitation in this study regarding the frequency of clinical assessments, which varied among patients. In our clinical protocol, we aimed for monthly examinations. Among the 36 responders, 29 patients (80.6%) were examined every month, four patients (11.1%) every 2 months, and three patients (8.3%) every 3 months. This discrete examination interval might have introduced a slight overestimation of the exact time to achieve clinical response in a small number of cases. Nevertheless, the substantial difference observed between the Th2-high and Th2-low groups (2.0 vs. 4.5 months) suggests that this assessment interval did not fundamentally alter the clinical implications of our findings.

These findings offer a critical warning for real-world clinical practice. If clinicians rigidly apply the conventional 3- to 4-month examination window, they risk misclassifying patients with Th2-low phenotype as “pseudo-non-responders.” Premature discontinuation would deprive these patients of a highly effective therapeutic option that simply requires more time to manifest its profound benefits.

In conclusion, the clinical manifestation of tezepelumab’s efficacy is significantly delayed in the Th2-low phenotype compared with the Th2-high phenotype. For the patients with Th2-low phenotype, physicians should avoid premature treatment withdrawal and maintain tezepelumab therapy for a minimum of 6 months to accurately assess its true, long-term clinical benefit.

## Article Information

### Author Contributions

Hideyasu Yamada conceptualized the study, performed the data analysis, and wrote the manuscript. Taisuke Nakaizumi contributed to data collection and critically reviewed the manuscript. Both authors approved the final version of the manuscript.

### Conflicts of Interest

Hideyasu Yamada has received honoraria for lectures from AstraZeneca and Sanofi. Taisuke Nakaizumi declares that he has no conflicts of interest regarding the publication of this article.

### Ethical Approval

This retrospective observational study was conducted in accordance with the Declaration of Helsinki. All participants provided written informed consent, and the local institutional review board approved the study protocols (IRB number: R01-350).
